# Lung recruitment: What has computed tomography taught us in the last decade?

**DOI:** 10.1186/s13613-019-0497-8

**Published:** 2019-01-22

**Authors:** D. Chiumello, P. Formenti, S. Coppola

**Affiliations:** 1 SC Anestesia e Rianimazione, Ospedale San Paolo – Polo Universitario, ASST Santi Paolo e Carlo, Via Di Rudinì, Milan, Italy; 2 Dipartimento di Scienze della Salute, Università degli Studi di Milano, Milan, Italy; 3 Centro ricerca coordinata di insufficienza respiratoria, Università degli Studi di Milano, Milan, Italy

## Abstract

Although chest X-ray remains a fundamental lung imaging technique, through the years, CT scan has significantly improved our knowledge of the pathophysiological process and currently is the reference lung imaging tool for both a visual and quantitative computer-based analysis. The application of lung CT in the early phase of ARDS has led to changes in the clinical management in up of thirty percent of the patients. Although CT requires the transportation of the patient to the radiological department and exposes the patient to high dose of radiation, given the several information that CT can offer, it should be applied at least one time, in the early phase in all ARDS patients. CT plays an irreplaceable role to describe and assess the lung recruitability and to help a more physiological setting of mechanical ventilation.

## Introduction

Acute respiratory distress syndrome (ARDS) is a clinical syndrome characterized by an inflammatory pulmonary edema, diffuse alveolar damage, increase in lung weight and decrease in the aerated lung regions [1]. The radiological hallmark of ARDS is the presence of bilateral pulmonary infiltrates on chest radiography without cardiovascular abnormalities [1]. Although chest X-ray remains a fundamental lung imaging technique, through the years, CT scan has significantly improved our knowledge of the pathophysiological process and currently is the reference lung imaging tool for both a visual and quantitative computer-based analysis [2]. It has been reported that the application of lung CT in the early phase of ARDS has led to changes in the clinical management in up of thirty percent of the patients [3]. Although CT requires the transportation of the patient to the radiological department and exposes the patient to high dose of radiation, given the several information that CT can offer, it should be applied at least one time, in the early phase in all ARDS patients.

## Lung recruitment

Typically, the distribution of the normally aerated lung regions, ground glass attenuation and consolidated regions in ARDS follows a ventral to dorsal gradient [2]. However, a similar sterno-vertebral distribution has been also described in cardiogenic pulmonary edema; suggesting that the type of lung insult, hydrostatic or inflammatory, does not influence the distribution of the resulting interstitial and intra-alveolar edema in ARDS and cardiopulmonary edema syndrome [4]. The amount of alveolar collapse mainly depends on the amount of lung edema, height of the lung and weight of the chest wall [5]. The most relevant information concerning lung recruitment has been derived from the quantitative analysis of lung CT. The effect of a recruitment maneuver in promoting the lung recruitment will depend both on the amount of applied pressure into the respiratory system and on the duration of the maneuver.

A recruitment maneuver, consisting of a PEEP level set at 10 cmH_2_O above the lower inflection point of the pressure volume curve applied for 15 min, significantly increases the recruited lung compared to a recruitment maneuver with a CPAP set at 40 cmH_2_O for 40 s both after 5 and 60 min [6]. A seminal study using a recruitment maneuver with an airway pressure of 45 cmH_2_O showed that in a large population of ARDS patients, the lung recruitability was quite heterogeneous ranging from 0 to 70% of the total lung weight (average lung recruitment 13%) [7]. De Mato et al. [8], by computing the lung recruitability as the weight of collapsed tissue that could be aerated from low to high PEEP level related to the total lung weight (similarly to the previous study), showed that the amount of non-aerated tissue decreased from 53 to 8% applying a recruitment maneuver consisting in plateau inspiratory pressure up to 60 cmH_2_O with a stepwise increments in PEEP levels from 10 to 45 cmH_2_O. This much higher lung recruitability, compared to the previous data, could be explained by a shorter time of ventilation (< 72 h of ARDS onset) and much higher positive fluid balance which could significantly increase the amount of lung edema and collapsed tissue with higher lung recruitability.

According to the Berlin definition, patients with severe ARDS had a significantly higher amount of lung edema, non-aerated lung tissue and potential for lung recruitment compared to the mild and moderate ARDS, and in fact, the mortality significantly increased with the ARDS severity assessed at 5 cmH_2_O of PEEP [9, 10]. The lung recruitment was poorly predictable being affected by the type of injury, amount of lung edema, timing of onset of ARDS and alteration in lung and chest compliance. However, among the different CT data, the lung recruitment was the only parameter independently associated with the outcome [10]. Differently, the amount of PEEP as the pressure necessary to maintain the lung completely opens to overcome the compressive force (lung and chest wall) was not related to the amount of recruitable lung tissue. In particular, the average PEEP to maintain open the lung was approximately 16 cmH_2_O. These data suggest that not only the amount, but also the distribution of lung edema (a prevalent intra-alveolar or interstitial edema) can significantly affect the lung recruitability. In fact, patients with a diffuse pattern compared to a lobar pattern usually present a higher lung recruitability.

This quantitative analysis of the lung recruitment by CT requires both a dedicated software and a manual delineation of all the lung CT slices with a total amount of work between 4 and 6 h. Consequently, this analysis remains only a research tool. An alternative to the anatomical analysis to assess lung recruitment could be a visual anatomical analysis. This simple analysis, which does not require any dedicated software, is feasible and had a good sensitivity and specificity to classify patients with high and low recruitability [11]. Moreover, in ARDS patients, the application of a low-dose chest CT scan with a reduction up to 30 mAs has been demonstrated to be a valuable tool for both a quantitative and visual analysis of the lung recruitment [12].

Alternative methods, to evaluate the lung recruitment, such as lung ultrasound or the pressure volume curve or changes in respiratory mechanics have been proposed. Regarding the lung ultrasound, Rouby et al. in ARDS patients showed a good correlation between the pressure volume curve and lung ultrasound in assessing the lung recruitability by PEEP modifications [13]. However, when lung ultrasound and CT scan were compared, they were not related [14]. In fact, the lung recruitment was assessed by quantitative CT as the decrease in non-aerated tissue and by lung ultrasound as the decrease in the global lung score. These apparently contradictory findings can be explained by the fact that the lung ultrasound score is the sum of different lung morphologies (from normal aeration to consolidation) and does not assess only the consolidated lung as CT does. Furthermore, the changes in lung gas volume assessed both by the pressure volume curve and by the changes in respiratory mechanics were not related to the lung recruitment computed by CT. These data suggest that variations in the gas volume can detect both an inflation of new alveolar units and further inflation of already open alveolar units, while CT scan evaluates only the inflation of new alveolar units. Similarly CT scan has taught us that hyperinflation refers to gas overfilling, whereas overdistention refers to the alveolar wall tension due to pressure, and consequently, the lung can be overdistended as in severe ARDS and not hyperinflated and vice versa as in emphysema [15].

In conclusion, CT plays an irreplaceable role to describe and assess the lung recruitability which is one the most important pathophysiological information to consider in the ventilatory management of ARDS patients.

## Abbreviations

ARDS: acute respiratory distress syndrome
CT: computed tomography
PEEP: positive end-expiratory pressure
